# Reframing Expectations about aging – Physical Activity and Inclusive Reappraisal (RE-PAIR): Protocol of a randomized intervention promoting positive self-perceptions of aging and physical activity in older couples

**DOI:** 10.1186/s12877-026-07603-7

**Published:** 2026-05-14

**Authors:** Serena Sabatini, Francesco Pagnini, Alice Martins Pederiva, Erik Henderson, Boris Cheval, Erika Borella, Vânia de la Fuente-Núñez, Denis Gerstorf, Fabrizio Mezza, Shevaun D. Neupert, Fiona S. Rupprecht, Feliciano Villar, Tim D. Windsor, Susanne Wurm, Maria Mataró

**Affiliations:** 1 Department of Clinical Psychology and Psychobiology, University of Barcelona, Campus Mundet, Ponent, Passeig de La Vall d’Hebron, 171, Barcelona, 08035 Spain; 2 School of Psychology, University of Surrey, Guildford, UK; 3 Department of Psychology, Università Cattolica del Sacro Cuore, Milan, Italy; 4 Univ Rennes, École Normale Supérieure de Rennes, VIPS2, Rennes, 35000 France; 5 Department of General Psychology, University of Padova, Padua, Italy; 6 IRCCS San Camillo Hospital, Venice, Italy; 7 Independent Researcher, Healthy Ageing and Ageism, Barcelona, Spain; 8 Department of Psychology, Humboldt University Berlin, Berlin, Germany; 9 Department of Humanities, Università Degli Studi Di Napoli Federico II, Naples, Italy; 10 Department of Psychology, North Carolina State University, Raleigh, USA; 11 Department of Psychology, University of Kaiserslautern-Landau, Landau, Germany; 12 Department of Cognition, Development and Educational Psychology, University of Barcelona, Barcelona, Spain; 13 College of Education, Psychology and Social Work, Flinders Institute for Mental Health and Wellbeing, Flinders University, Adelaide, Australia; 14 Department of Prevention Research and Social Medicine, Institute for Community Medicine, University of Greifswald, Greifswald, Germany; 15 Institut de Neurociències, Universitat de Barcelona, Barcelona, Spain; 16 Institut de Recerca Sant Joan de Déu, Esplugues de Llobregat, Spain

**Keywords:** Spouses, Partners, Views on aging, Age stereotypes, Subjective aging, Psychoeducational intervention, Physical activity intervention, Behavioral intervention

## Abstract

**Background:**

Ageism and negative self-perceptions of aging have a detrimental impact on the health of older individuals, and on the healthcare and economic systems. Although existing psychoeducational and behavioral interventions have effectively increased positive self-perceptions of aging, often their beneficial effects have been investigated with short follow-ups or faded over time. This may be due to interventions having been delivered at the individual level without sufficient attention to social context, but individuals’ self-perceptions of aging are likely influenced by the age-related beliefs and behaviors of people close to them.

**Objective:**

We aim to deliver a 12-week multicomponent intervention promoting positive self-perceptions of aging and physical activity to investigate (1) whether it promotes significant change in primary (i.e., self-perceptions of aging and physical activity) and secondary (i.e., self-directed and other-directed ageism, subjective views of couple’s joint aging, perceived age-related changes in one’s partner, anxiety and depressive symptoms, physical fitness, and executive function) intervention outcomes; (2) whether observed change in intervention outcomes is greater when the intervention is delivered to couples compared to when it is delivered to one partner alone; (3) the extent to which the effect of the intervention is extended from one partner who undertakes the intervention to the other partner who does not undertake it.

**Methods:**

We will adopt a three-arm randomized controlled trial (RCT) design with pre, immediate post-intervention, and three- and six-months follow-up assessments. The sample will be divided into three groups, each comprising 60 spouses, cohabiting partners, or long-standing partners aged ≥ 65 years. In the first group, partners will undertake together the intervention, comprising a psychoeducational and a behavioral component. In the second group, only one partner will undertake the same intervention. In the third/control group none of the partners will undertake the intervention during data collection. Self-perceptions of aging, physical activity, ageism, subjective views of couple’s joint aging, perceived age-related changes in one’s partner, and anxiety and depressive symptoms will be assessed via questionnaires whereas physical fitness, executive function, and attention will be objectively assessed in person.

**Conclusion:**

This project will provide evidence on whether targeting couples could enhance/facilitate promotion of positive self-perceptions of aging and physical activity in older age.

**Trial registration:**

ClinicalTrials.gov: NCT07113860. Release date: 03–07–2025.

Protocol version 3: 1–3–2026.

**Supplementary Information:**

The online version contains supplementary material available at 10.1186/s12877-026-07603-7.

## Introduction

The global proportion of older people (i.e., aged ≥ 65 years) is rapidly increasing. According to the United Nations, worldwide there are 830 million people aged ≥ 65 years, and this number is projected to reach 1.7 billion by 2054 [1]. Of all the continents, Europe has, and is expected to continue to have, the largest proportion of people ≥ 65 years. In Spain alone in 2024 there were 9.93 million people aged ≥ 65 years, which equates to one-fifth of the Spanish population [2]. As the proportion of older people in the population grows, the number of individuals living with chronic health conditions is also rising, reflecting the increased prevalence of such conditions in later life. Based on the Survey on Health, Aging and Retirement in Europe, 36% of people ≥ 65 years reported having at least two chronic diseases across EU countries in 2020. In Spain, it is estimated that about 60% of people ≥ 65 years have at least one chronic health condition and 40% of men and 44% of women aged ≥ 65 years have multiple chronic health conditions [3]. This can impact people’s quality of life, the capacity of older adults to remain socially engaged, and healthcare and economic systems.

Finding new ways to promote active and healthy aging is a global research priority [4]. Healthy aging has been defined by the World Health Organization as “the process of developing and maintaining the functional ability that enables well-being in older age” ([5], p.28). Numeorus modifiable factors contribute to healthy aging [6] including improvements in the health care system [7, 8], the environment [9, 10], and in one’s behavior, as well as in beliefs and attitudes towards health-related behaviors and health. Accumulating evidence suggests that individuals’ self-perceptions of aging may be an additional potentially modifiable risk factor for inactivity, poor health, and earlier mortality in older age [11, 12]. More specifically, a recent meta-analysis found that self-perceptions of aging have a small effect on health and mortality (i.e., Pearson’s correlation coefficient of 0.12) [13].

Self-perceptions of aging refer to experiences and perceptions that people have of their own aging [14]. While some measures assessing self-perceptions of aging capture individuals’ global perception of their own aging [15], other measures can capture a mix of positive and negative experiences that individuals can have across several life domains (e.g., physical health, mental health, cognition, lifestyle, etc.) [16]. Examples of negative self-perceptions of aging are noticing age-related limitations and health issues, whereas examples of positive self-perceptions of aging are being grateful for aging-related benefits such as having greater freedom and knowledge. Typically, middle-aged and older adults have a mix of positive and negative self-perceptions of aging, with negative self-perceptions of aging increasing with age [17, 18]. In midlife and old age individuals with more positive self-perceptions of aging consistently show greater maintenance of mental, physical, and cognitive health [13] and greater quality of life [19]. In contrast, individuals with more negative self-perceptions of aging have higher risks of depressive and anxiety symptoms, cognitive decline and dementia, physical conditions, and other health issues such as poor sleep and pain [13, 20–24]. In the United States alone, Levy and colleagues estimated that the annual healthcare expenditures due to negative self-perceptions of aging is $33.7 billion [25]. Finally, a recent meta-analysis showed that people with more negative self-perceptions of aging are at greater risk of mortality (small effect) [13].

Individuals’ positive and negative experiences of aging may be influenced by how they are perceived and treated by relevant others and the society (i.e., ageism). The term ageism refers to individuals’ beliefs (i.e., stereotypes), feelings (i.e., prejudice) and behaviors (i.e., discrimination) about older people and aging in general. Importantly, ageism can not only be applied towards others (i.e., other-directed ageism) but also towards the self (i.e., self-directed ageism). An example of self-directed ageism could be an older person that decides to avoid learning how to use a smartphone because they think they are too old for technologies. In the context of this project, it is essential to note that self-perceptions of aging are conceptually different from self-directed ageism. Indeed, even though some aspects of self-directed ageism may overlap with self-perceptions of aging and self-perceptions of aging may somewhat be influenced by ageism, self-perceptions of aging comprise both positive and negative self-perceptions that are based on individuals’ own life experiences rather than solely on general and negative stereotypes. An analysis of data from 83,000 participants from 57 countries reported in the WHO Global report on Ageism found that ageism -including the dimensions of stereotypes and prejudice- against older people was present in 55.5% of the global population and 44.2% of the European population [26]. In Spain, an analysis of 501 headlines across two national newspapers revealed that 71% of these portrayed older people in a negative way [27]. In sum, estimations suggest that half of the population may hold ageist attitudes towards older people. This is alarming as ageism may promote and reinforce negative self-perceptions of aging [28], and negative self-perceptions of aging are detrimental for individuals’ health [13, 25].

The detrimental effects of ageism and negative self-perceptions of aging on health outcomes can be explained by Levy’s Stereotype Embodiment Theory [29, 30]. According to this theory, and supporting evidence, ageism and self-perceptions of aging influence health through three pathways: psychological, behavioral, and physiological. The psychological pathway captures how ageism and negative self-perceptions of aging undermine positive psychological characteristics such as self-efficacy, will to live, and a positive outlook toward the future [31–33]. The behavioral pathway reflects how ageism and negative self-perceptions of aging cause disengagement from health-enhancing and adaptive behaviors (e.g., physical activity and taking prescribed medications) [11, 34–37]. Lastly, the physiological pathway captures how ageism and negative self-perceptions of aging can lead to negative biological processes (e.g., increases in biomarkers of inflammation such as C-reactive protein) and, consequently, to health-related conditions [38–40]. However, one could hypothesize that some of these paths are intertwined. For example, individuals with higher perceived control over their health and aging (psychological pathway) may be more engaged in physical activity (behavioral pathway) and individuals who engage more in physical activity may experience greater regulation of the inflammatory system (physiological pathway) [41, 42].

Overall, given the presumed undermining effects that ageism have on people’s self-perceptions of aging, and the detrimental impact that both ageism and self-perceptions of aging may have on people’s health [25, 43], the widespread presence of ageism and negative self-perceptions of aging can have massive consequences on older people’s lives, and healthcare and economic systems. Consequently, finding strategies to reduce ageism and to promote accurate knowledge about aging and positive self-perceptions of aging is a global priority [44]. In this regard, over the past few years, several intergenerational programs and psychoeducational interventions have been developed with the aim to increase accurate knowledge about aging and to promote more positive self-perceptions of aging [28, 45–49]. Behavioral programs, promoting engagement with physical activity, were also found as an effective means to promote positive self-perceptions of aging [37]. Finally, multidimensional interventions comprising both a psychoeducational and a behavioral component (promoting physical activity) were also found effective [50, 51]. More specifically, a recent meta-analysis of 12 studies found that the impact of existing interventions on self-perceptions of aging has a moderate effect size (i.e., Hedges’s g of − 0.56; 95% CI: − 1.06 to − 0.07, *p* = 0.03; equating to a Pearsons’ correlation coefficient of −0.27) [46]. Moreover, these interventions were not only effective in increasing positive self-perceptions of aging, but they also led to benefits in participants’ physical functioning and mood. Although there is the need of more evidence comparing the effectiveness of different types of interventions, perhaps interventions that comprise both a psychoeducational and a behavioral component may be particularly effective as both means can promote positive self-perceptions of aging and both positive self-perceptions of aging and physical activity typically lead to health benefits [13, 46, 52, 53].

One limitation of some psychoeducational and behavioral interventions promoting positive self-perceptions of aging is that their positive effects seem to fade over time (e.g., [51]). It may be that the dwindling efficacy of these interventions is because they have been delivered at the individual level. In contrast, it stands to reason that individuals’ knowledge about aging and self-perceptions of aging are influenced by the beliefs that people close to them have about aging [54–56] and by how they are treated by others during daily interactions and conversations [57]. For example, being treated with little respect (e.g., being ignored during conversations) and/or receiving remarks highlighting one’s age-related losses (e.g., forgetfulness) may make older people feel negative about themselves, their relationships to others, and their aging process [28]. In support of this reasoning, previous evidence suggests that older people often change their priorities and give up pleasant activities according to what the society and their social network consider appropriate for their age [58], and one’s behaviors can then shape self-perceptions of aging [51]. For example, a common misconception is that older people cannot or should not engage in high intensity physical activity [52, 59]. In conclusion, if the aim is to change how people think about themselves, it may also be necessary to modify how they are perceived and treated by other people, especially relevant others.

Among social bonds, spouses/partners may have a particular influence on each other’s knowledge about aging and self-perceptions of aging. Cross-sectional and longitudinal studies have indeed reported small-to-moderate associations between the self-perceptions of aging of spouses and have shown that spouses can influence each other’s self-perceptions of aging over time [54, 56, 60–62]. According to the Developmental-Contextual Model of Couple Synchrony Across Adulthood and Old Age [63] similarity in partners’ self-perceptions of aging may be due to partners over time becoming more and more similar in their behaviors and beliefs due to closeness, shared contexts, and shared life events. This interpretation would also be in line with Interdependence Theory [64], which postulates that long-term partners influence each other’s needs, thoughts, and motives, everyday behaviors, physiological characteristics, and health and well-being. Furthermore, changing individuals’ self-perceptions about aging may not only improve psychosocial outcomes but could also impact biological processes via placebo-like mechanisms, as shown in recent studies on mind–body expectations (e.g., [65]). In line with these results, psychological reappraisal could serve as a low-cost lever for physical health in aging populations (e.g., [65]).

### The current study

The current study will adopt a three-arm randomized controlled trial (RCT) design with pre- and post-intervention assessment. A multicomponent (psychoeducational and behavioral) intervention called RE-PAIR (Reframing Expectations about aging – Physical Activity and Inclusive Reappraisal) and aiming to promote, as primary outcomes, positive self-perceptions of aging and walking in older couples (i.e., spouses, cohabiting partners, or long-standing non-cohabiting partners) will be delivered to three groups. In Group 1, hereafter referred to as *dual-partner intervention group*, both partners will undertake the intervention together. In Group 2, hereafter referred to as *single-partner intervention group*, only one of the two partners will undertake the intervention. In Group 3, hereafter referred to as *control group*, none of the partners will undertake the intervention during data collection. Participants allocation to intervention groups will be on a 1:1:1 ratio.

**Fig. 1:**
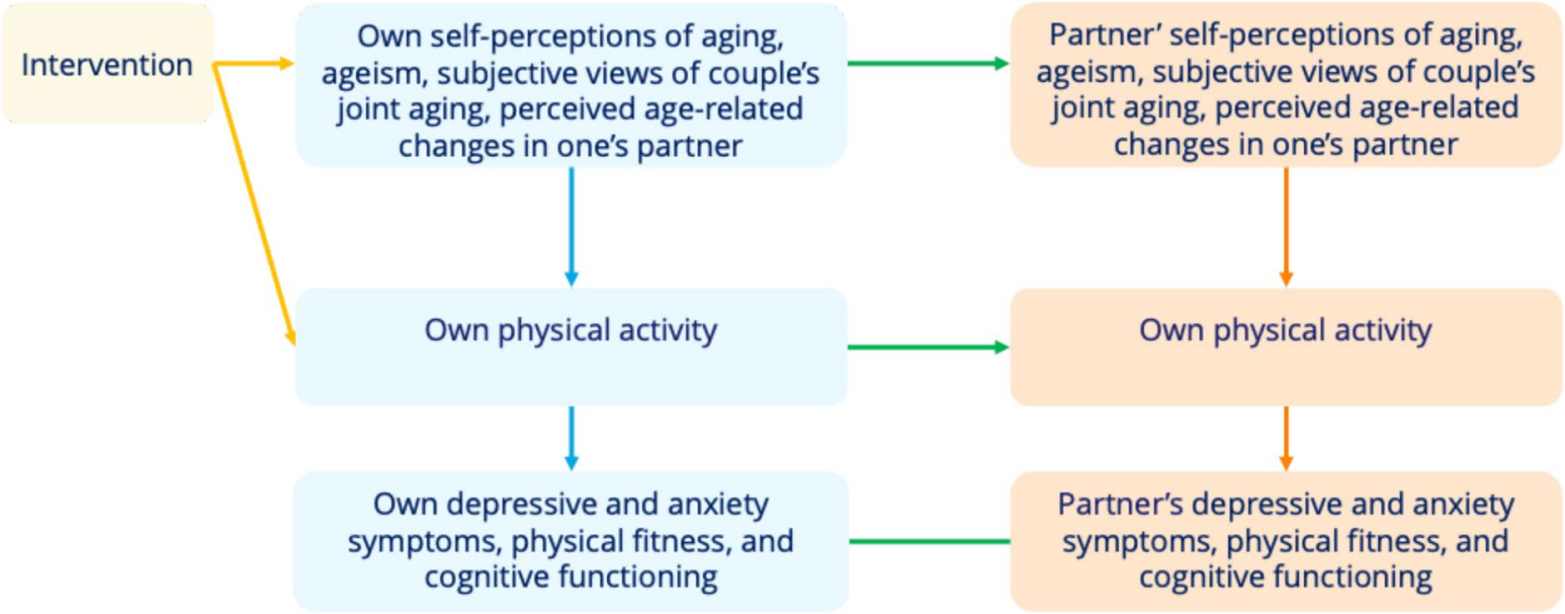
Dyadic effects of the multi-component intervention

Primary intervention outcomes are changes in self-perceptions of aging and in physical activity engagement, reflecting proximal targets of intervention. That is, participants’ scores on the perceived age-related gains and losses subscales of the awareness of age-related change questionnaire [16], felt age [15], subjective nearness to death [66], the international physical activity questionnaire short form [67], and the Saltin-Grimby physical activity level scale [68, 69] (See Fig. 1). Secondary intervention outcomes are changes in participants’ scores on the experienced ageism questionnaire [70], the other-directed ageism questionnaire [70], subjective views of couple’s joint aging [71], perceived age-related gains and losses in one’s partner assessed with the adapted informant-version of the awareness of age-related change questionnaire [16], patient health questionnaire [72], senior fitness test [73], grip strength [74], and a composite score for executive function (See Table 1). Change in ageism is treated as a secondary outcome to explore whether shifts in self-perceptions of aging are accompanied by changes in ageism, without conflating the constructs.

**Table 1.** Study hypotheses and explorative questions

Hypotheses addressed using quantitative data	
Hypothesis 1	Relative to those in the control group, between pre- and post- intervention, participants who undertake the multicomponent intervention (dual-partner intervention group and half of single-partner intervention group) will experience the following changes in primary intervention outcomes: a greater increase in perceived age-related gains [16], a greater decrease in perceived age-related losses [16], a younger felt age [15], lower subjective nearness to death, higher scores on the international physical activity questionnaire short form [67], and higher scores the Saltin-Grimby physical activity level scale [68, 69]. In a more exploratory way it is also expected that, relative to those in the control group, between pre- and post- intervention, participants who undertake the multicomponent intervention (dual-partner intervention group and half of single-partner intervention group) will report less experienced ageism [70], less other directed ageism [70], a more positive subjective view of the couple’s aging [71], higher perceived age-related gains in one’s partner [16], lower perceived age-related changes in one’s partner [16], fewer depressive symptoms in the patient health questionnaire [72], greater physical fitness in the senior fitness test [73], greater grip strength [74], and greater executive function (i.e., composite score based on participants’ standardized scores on the trail making test [75], the digit span backward task [76], the PMR phonological verbal fluency test, the animal naming fluency test [77], the Stroop color-word test [78], and the D2 test [79]). Moreover, it is expected that these changes are better maintained over time among participants who undertake the multicomponent intervention compared to the control group
Hypothesis 2	It is hypothesized that, compared to when delivering the intervention to only one partner (single-partner intervention group), delivering it to both partners (dual-partner intervention group) will lead to a greater and more lasting increase in perceived age-related gains [16], decrease in perceived age-related losses [16], increase in younger felt age [15], decrease in subjective nearness to death, increase in scores on the international physical activity questionnaire short form [67], increase in scores on the Saltin-Grimby physical activity level scale [68, 69]. In a more exploratory way, it is also expected that, compared to when delivering the intervention to only one partner (single-partner intervention group), delivering it to both partners (dual-partner intervention group) will lead to a greater and more lasting decrease in experienced ageism [70], decrease in other directed ageism [70], increase in positive subjective view of the couple’s aging [71], increase in perceived age-related gains in one’s partner [16], decrease in perceived age-related changes in one’s partner [16], decrease in depressive symptoms in the patient health questionnaire [72], increase in scores on the senior fitness test [73], increase in grip strength [74], and increase in executive function (i.e., composite score based on participants’ standardized scores on the trail making test [75], the digit span backward task [76], the PMR phonological verbal fluency test, the animal naming fluency test [77], the Stroop color-word test [78], and the D2 test [79]
Hypothesis 3	In the single-partner intervention group, participants increase in perceived age-related gains [16], decrease in perceived age-related losses [16], increase in younger felt age [15], decrease in subjective nearness to death, increase in scores on the international physical activity questionnaire short form [67], increase in scores on the Saltin-Grimby physical activity level scale [68, 69] is observed at least to a small extent in the other partner In a more exploratory way, it is also expected that, in the single-partner intervention group, decrease in experienced ageism [70], decrease in other directed ageism [70], increase in positive subjective view of the couple’s aging [71], increase in perceived age-related gains in one’s partner [16], decrease in perceived age-related changes in one’s partner [16], decrease in depressive symptoms in the patient health questionnaire [72], increase in scores on the senior fitness test [73], increase in grip strength [74], and increase in executive function (i.e., composite score based on participants’ standardized scores on the trail making test [75], the digit span backward task [76], the PMR phonological verbal fluency test, the animal naming fluency test [77], the Stroop color-word test [78], and the D2 test [79] is observed at least to a small extent in the other partner

Research questions addressed using qualitative data	
Research question 1	How do participants in the dual-partner intervention group engage with the intervention tasks, and to what extent do they undertake these tasks jointly as a couple?
Research question 2	In what ways do participants in the single-partner intervention group involve their non-participating partner in the intervention activities?
Research question 3	What changes do participants in the dual-partner intervention group and single-partner intervention group report in their self-perceptions of aging, as well as in their perceptions of and interactions with their partner?

Quantitative data collected through this study design will make it possible to test three main hypotheses. First, participants who undertake the multicomponent intervention (dual-partner intervention group and half of single-partner intervention group) will experience changes in primary and secondary intervention outcomes between pre- and post-intervention and these changes will be larger and better maintained at follow-ups relative to those in the control group [50, 53]. Expected change in self-perceptions of aging is based on previous research that tested the efficacy of the same psychoeducational component that will be used in this study [50, 53]. Similarly, expected change in physical activity is based on previous research that tested the efficacy of the behavioral component used in this study [80]. However, psychoeducational interventions targeting views on aging have also been found effective to promote physical activity [45, 46, 49]. It is expected that the intervention will decrease ageism as participants will be thought to disregard age-stereotypes. Change in anxiety and depressive symptoms and physical fitness is expected as a recent meta-analysis of 12 studies found that self-perceptions of aging interventions led to improvements in physical performance and mental health among older adults [46]. The remaining secondary outcomes (subjective views of couple’s joint aging, perceived age-related gains and losses in one’s partner, and executive functions) have never been explored as outcomes of self-perceptions of aging interventions and will therefore be a novel component of this project. Nonetheless, given the associations that have been reported between partners’ self-perceptions of aging [54, 56, 60–62], it is expected that the multicomponent intervention will lead to changes in how participants perceive the aging of their partner and of their couple. It is hypothesized that the multicomponent intervention will lead to a change in cognitive functioning as the behavioral component of the intervention has been previously shown effective to increase cognitive abilities (e.g., working memory, attention, and processing speed) [80]. Moreover, self-perceptions of aging have been found associated with cognitive functioning over time [23, 24, 81]. Second, based on previous evidence on physical activity [82], it is hypothesized that compared to when delivering the intervention to only one partner (single-partner intervention group), delivering it to both partners (dual-partner intervention group) will lead to a greater and more lasting change in primary and secondary intervention outcomes. Third, in the single-partner intervention group, participants change in intervention outcomes is observed at least to a small extent in the other partner due to partners tending to influence each other’s beliefs and behaviors [83]. More details on the expected changes for study outcomes are reported in Table 1. Finally, qualitative data collected at the end of the intervention through interviews of ten couples from each of the dual-partner intervention group and the single-partner intervention group will make it possible to test three additional research questions, outlined in Table 1.

## Methods

### Study oversight and schedule

The Reframing Expectations about aging – Physical Activity and Inclusive Reappraisal (RE-PAIR) project is a single-center three-arms randomized pre- post-intervention design that will take place mainly at the University of Barcelona (Barcelona, Spain), in the Department of Clinical Psychology and Psychobiology, and in collaborating centers (e.g., Espai Mataró Cuida-Care CityLab). This protocol was developed in accordance with the Standard Protocol Items Recommendations for Interventional Trials (SPIRIT) 2013 Statement [84] (See supplementary Text 1). The intervention components are described using the Template for Intervention Description and Replication (TIDieR) checklist to ensure transparency and replicability [85]. Moreover, this project will be run in compliance with the Bioethics Commission of the University of Barcelona from which ethical approval was obtained (Institutional Review Board IRB00003099 CER092416). Eventual amendments to aspects of the project design will be submitted for further review by the Bioethics Commission of the University of Barcelona and followed by protocol amendments in Clinicaltrials.gov.

**Fig. 2:**
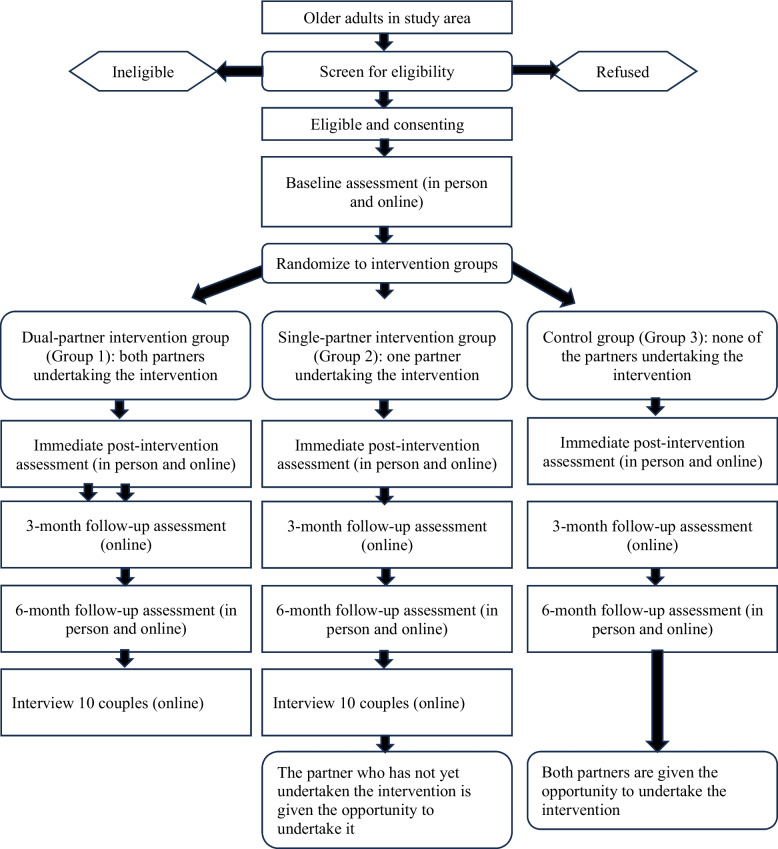
Study design

Figure 2 illustrates the process for data collection and Table 2 reports a summary of study measures and describes at which timepoints they will be administered. After having obtained participants’ written informed consent in person, baseline, immediately post-intervention, and six-month follow-up assessments will be conducted as one-on-one sessions in the facilities of the Department of Clinical Psychology and Psychobiology or collaborating centers (e.g., Espai Mataró Cuida-Care CityLab). During these assessments, participants will also independently complete some online questionnaires after having been shown by the researcher how to fill the online questionnaire and having been given the opportunity to ask technology related questions. The three-month follow-up assessment will instead be conducted online only. The sessions of the psychoeducational component of the intervention will be delivered in small‑group sessions held primarily in a seminar room of the Department of Clinical Psychology and Psychobiology at the University of Barcelona. To ensure fidelity the psychoeducational intervention sessions will always be delivered by the same researcher (principal investigator) while adhering to the intervention manual. When required for logistical reasons, equivalent facilities at collaborating centers (e.g., Espai Mataró Cuida-Care CityLab) will be used, following the same procedures and materials. The behavioral component of the intervention will be undertaken independently by participants after having received in person instructions by the researchers during the first psychoeducational session.

**Table 2:** Timeline of measures administered throughout the randomized clinical trial

Construct	Measure	Baseline	Intervention	Post-intervention	3-month follow-up	6-month follow-up	Reference
Physical activity	International Physical Activity Questionnaire short form	X		X	X	X	[67]
	Saltin-Grimby Physical Activity Level Scale	X		X	X	X	[68, 69]
	Daily diaries		X				
	Borg Rating of Perceived Exertion scale		X				[86]
Self-Perceptions of Aging	Awareness of Age-Related Changes 10-SF self-administered version	X		X	X	X	[16, 87]
	Felt age	X		X	X	X	[15]
	Subjective nearness to death	X		X	X	X	
	Subjective Views of Couple's Joint Aging	X		X	X	X	[71]
	Subjective Aging Perception Scale	X					[88, 89]
Ageism	World Health Organization ageism scale	X		X	X	X	[70]
	Expectations Regarding Aging	X					[90]
Cognitive functioning	Trail Making Test A and B	X		X		X	[75]
	Digit Span Forward and Backward	X		X		X	[76]
	PMR Phonological Verbal Fluency Test	X		X		X	[77]
	Animal Naming Fluency Test	X		X		X	[77]
	Stroop Color-Word Test	X		X		X	[78]
	D2 test	X		X		X	[79]
Physical fitness	Grip strength assessed with a dynamometer	X		X		X	[74]
	Senior Fitness Test	X		X		X	[73]
	Body mass index assessed through self-reported weight and height	X		X		X	
	Waist circumference (assessed by a researcher)	X		X		X	
Physical health	List of physical health conditions	X		X	X	X	
Mental health	Patient Health Questionnaire	X		X	X	X	[72]
	List of mental health conditions	X		X	X	X	
Couple Dynamics	Quality of Marriage Index	X		X	X	X	[91]
	Single-item assessing dominance	X		X	X	X	[92]
	Awareness of Age-Related Changes 10-SF observant version	X		X	X	X	[16, 87]
	Length of couple relationship; presence of children and/or grandchildren	X					
Sociodemographic Questions	Age; sex; gender; sexual orientation; ethnic origin; education; marital status; employment status; income	X					
Social status	Subjective Social Status	X					[93]
Personality	Ten-Item-Personality Inventory	X					[94]

### Sample size

This study aims to recruit 360 participants (i.e., 180 couples), randomly allocating 120 individuals (i.e., 60 couples) to each of the three intervention groups. To address the first two research questions, we will compare two groups in the analyses: first (dual-partner intervention group) and second (single-partner intervention group) groups combined versus the third (control) group for the first hypothesis and the first (dual-partner intervention group) versus the second (single-partner intervention group) group for the second hypothesis. To detect a small-to-moderate effect as Cohen’s f = 0.25 (corresponding to a Pearsons’ correlation coefficient of 0.24) with power = 0.80 and considering two groups and four measurement points, we will need 96 individuals per group. Considering an expected attrition of 20%, we will need 120 individuals (60 couples) for each group, leading to 360 individuals/participants (180 couples) in total. A 20% drop out of participants is expected due to similar percentages having been reported in previous behavioral interventions with similar length of follow-up [95, 96]. To address the third research question, we plan to use latent change score models across four measurement point. In this regard, a sample comprising 360 will be sufficient to detect a moderate effect.

### Recruitment

It is expected that recruitment will last at least 12 months (between July 2025 and July 2026). Participants will be recruited in Barcelona via flyers, newsletters, and presentations at civic centers, senior centers, libraries, and local organizations such as Amics de la Gent Gran; Fundació Catalunya La Pedrera; Universitat de l’experiència; Federacio d’associacions de gent gran de Catalunya; IDIAP Jordi Gol Fundació Institut Universitari per a la Recerca a l'Atenció Primària de Salut Jordi Gol i Gurina. Primary care centers will also be contacted to recruit participants. Presentations will be delivered by the principal investigator (Dr Serena Sabatini) and a PhD candidate in Psychology. Participants will also be recruited through social media such as X, Facebook, Instagram, and LinkedIn. Snowballing technique will also be used. Should recruitment be slow, sources of recruitment will be diversified through contact of associations outside Barcelona (Table 3).

**Table 3:** Summary of content of psychoeducational intervention session

Session	Content
1	Quiz on aging to detect own negative self-perceptions of aging as well as misconceptions regarding age and aging. Possible false beliefs are corrected by providing information on empirical findings during the discussion of the answers
2	Provision of information and group discussion on benefits of more positive self-perceptions of aging regarding health, functional abilities, cognitive abilities, and longevity. Participants are given homework for the next intervention unit: to observe themselves or others to detect negative age stereotypes or self-perceptions of aging in everyday life and to write down the situations and the negative self-perceptions of aging on a worksheet
3	Session two homework is presented and discussed in the group. A technique to change negative automatic thoughts about aging [97] is taught. During the session, participants will be given some time to apply this technique to their own situations recorded in their last homework, and this will be followed by a group discussion

### Inclusion and exclusion criteria

Individuals’ eligibility (i.e., inclusion and exclusion criteria) will be screened via phone (10—15 min). The full list of screening questions can be found in Supplementary Text. Inclusion criteria are: (1) living in the community in Barcelona or nearby areas; (2) both partners being aged ≥ 65 years; (3) speaking fluent Catalan or Spanish (judgment made by the researcher, who is a clinical psychologist, during the screening call); (4) being married, having a partner with whom they co-habit, or having had a partner for at least one year and with whom they meet at least twice a week. This criterion aims to ensure a stable relationship while maintaining recruitment feasibility. (5) having access to a smartphone, tablet, laptop, or computer with internet; (6) both partners being willing to participate in the study and providing written informed consent. Although the use of a chronological age cut-off to define older people is inherently arbitrary, this study adopts the threshold of 65 years and older to establish inclusion criteria for participation. This decision reflects the increasing self-relevance of age-stereotypes and self-perceptions of aging in later life, particularly among the oldest age groups [29, 98]. Participants can either possess a device that can be connected to the internet or use the device of a family member, neighbor, or friend. Moreover, participants with low technology literacy can nonetheless take part in the study as during baseline assessment the research team will show them how to complete the online questionnaires. Moreover, the research team will be available via email and phone to answer participants’ queries related to completion of the online questionnaires. Both retired and working people are eligible to take part in the study. People of any race can take part in the study. Couples of all sexual orientations are welcome to participate in the study.

Exclusion criteria are: (1) having a self-reported or clinically diagnosed neurodegenerative disorder, and/or an Adult Lifestyles and Function Interview Mini Mental State Examination (ALFI-MMSE) score of < 15 out of 22, indicating mild cognitive impairment or dementia [99, 100]. The research team has considerable experience with the administration of the MMSE and will adjust scores for individuals with low cultural background; (2) currently having a clinical diagnosis of mental health disorder that significantly impacts on the daily functioning of the individual (i.e., a diagnosis that significantly impacts the participants ability to work, do house chores, and/or engage socially; see Open Science Framework for more details [101]); (3) current substance use disorder (alcohol or drug abuse), or a history of substance abuse within the past five years that may interfere with study participation or adherence to the intervention; (4) having a physical condition or physical limitations that impair from starting a physical activity intervention consisting in walking between three to five times per week and to do some physical tasks such as stand up from and sit down on a chair and back scratch; (5) uncorrected severe hearing or visual impairment that would preclude following the intervention materials or instructions. Physical limitations will be self-reported by participants via phone but also further objectively assessed by the researchers at baseline assessment. Individuals with neurodegenerative disorders will be excluded as it could be difficult for them to understand and participate in the psychoeducational sessions, and/or engage in brisk walking. Individuals with current severe mental health disorders and/or with substance abuse will be excluded as these groups are less likely to be compliant with intervention conditions. However, individuals with more common mental health disorders (i.e., depressive disorders and anxiety disorders) that are not severe enough to significantly impact the daily functioning of the individual will be allowed to take part in the study. Individuals with significant physical limitations will be excluded as they could put themselves at risk of falling while engaging in physical activity/walking.

### Study design

Data collection is expected to start in September 2025 and to end at the end of May 2027. Data collection will take place in sequential phases, as it will start each time a group of approximately 42 participants (21 couples) will have been recruited. This group will then be further divided into the three intervention groups. Those who fulfill inclusion criteria will be invited to take part in the study via email, phone text, and/or phone call. Baseline assessment will comprise an in-person assessment of 60 min at the University of Barcelona or collaborating centers and an online survey created using Microsoft forms. Starting from the in-person assessment, the researcher will first go through the information sheet with the participant and will ask the participant to sign their informed written consent (about 10 min). A sample of information sheet and consent form can be found in Open Science Framework [101]. Second, participants will undertake paper and pencil cognitive tasks (about 10 min). Third, participants will undertake a 15-min physical fitness test and their waist circumference will be taken by the researcher. Fourth, using a tablet/computer facilitated by the researcher, participants will be asked to complete the first part of an online survey comprising questions on sociodemographic information, their physical and mental health, their engagement in physical activities, social network, loneliness, intergenerational contact, and aspects of their relationship with their partner (taking approximately 25 min). Participants will then be asked to complete within 24 h the second part of the online survey comprising questions about self-perceptions of aging; ageism; subjective views of couple's joint aging; personality traits; quality of marriage; and perceived age-related changes in one’s partner (taking about one hour). Couples will then be randomly allocated by a facilitator to one of the three intervention groups using a statistical software (i.e., STATA). Group allocation will be communicated to participants via email and/or text message after baseline assessments are completed. This communication will be sent by a member of the research team (facilitator) that does not assess participants nor delivers the intervention. Everyone from the dual-partner intervention group and half from the single-partner intervention group will undertake the intervention at the University of Barcelona or collaborating centers (e.g., Espai Mataró Cuida-Care CityLab). Individuals of the dual partner-intervention group will be assigned to the same intervention class as their partner. More specifically, the dual-partner intervention group will be further divided into nine classes (six classes comprising 14 individuals and three classes comprising 12 individuals). Those from the single-partner intervention group who will undertake the intervention will be further divided into five classes (five classes of 12 people each). The partner from the single-partner intervention group who will receive the intervention will be randomly selected while taking into account age and gender. For practical reasons, the principal investigator will deliver the intervention and therefore cannot be blinded to group allocation. Randomization will be computer-generated by an independent researcher and communicated only for scheduling purposes. Outcome assessors will remain blinded, and data will be analyzed from a de-identified dataset with masked group labels (A/B) following the preregistered analysis plan. Group identity will be revealed only after completion of the primary analysis. Moreover, participants will be instructed not to disclose their group allocation to the assessors during post‑intervention.

The control group will purposefully not receive an alternative intervention as this study aims to test the effects of the intervention compared to undertaking none. The control group will not receive any intervention during the study period to assess natural changes in couples’ perceptions and behaviors over time. This design enables comparison between participants’ trajectories in their normal environment and those exposed to the psychoeducational program. While this passive control condition does not control for nonspecific factors such as social engagement or group interaction, these have been addressed in previous trials validating the group-based format [50, 51]. The potential influence of such factors will be considered as a study limitation. During the 12-week intervention, participants from all groups will be asked to record in a-priori form every time they engage in physical activity. In doing so participants will also have to record how much and at which intensity—using the Borg Rating of Perceived Exertion Scale [86]—they engaged in physical activity. During the last session of the in-person intervention, participants will be asked to complete a paper and pencil questionnaire comprising a mix of multiple answers and open-ended questions (18 questions taking about 15 min) covering perceived utility and pleasantness of the intervention and whether and how they feel they have changed how they perceive themselves and their partner. In addition, participants from the dual-partner intervention group will be asked whether they think their partner sees/treats them differently post-intervention, compared to pre-intervention, and about their experience of having undertaken the intervention with their partner. Participants from the single-partner intervention group will instead be asked whether and how they think their partner (who did not undertake the intervention) benefitted from their participation to the intervention, and about their experience of undertaking the intervention without their partner. Participants from all groups will be assessed again both in person and through self-administered online questionnaires at post-intervention and at six-months follow-ups. All participants will also be assessed through self-administered online questionnaires at three-month follow-up. In person they will complete the same cognitive tasks and physical fitness tests as at baseline assessment. Online they will complete a selection of baseline questionnaires assessing eventual changes in demographic information and physical and mental health; self-perceptions of aging; engagement in physical activities; ageism; subjective views of couple's joint aging; quality of marriage; and perceived age-related changes in their partner. In the two weeks following the last session of the intervention ten couples from the double-partner intervention group and ten couples from the single-partner intervention group will also undertake a 20-min online interview using Phone/Teams/Zoom. Partners will be interviewed separately. Once data collection has ended, the remaining half of the single-partner intervention group and everyone from the control group will be offered the intervention as a big group.

Each participant will receive a total of €50 for completing all phases of the study (€100 per couple). Two older people who are not part of the project sample went through the material of the psychoeducational intervention, the surveys, and interviews scripts. Each of these individuals was paid €75 for their time.

## Measurements

Study questionnaires and interview scripts can be found in Open Science Framework [101].

### Primary intervention outcomes

#### Self-perceptions of aging

Outcomes assessing self-perceptions of aging will be the Awareness of Age-Related Change questionnaire, a one-item question assessing Felt Age, and a single-item assessing participants’ Subjective Nearness to Death [66]. The combination of these three different measures will be used to provide a comprehensive assessment of self-perceptions of aging.

The *Awareness of Age-Related Change* (AARC) 10-item questionnaire [16, 87] assesses perceived age-related gains and losses. Each of the items of the perceived age-related gains and losses subscales represents one of the five AARC behavioral domains that are health and physical functioning; cognitive functioning; interpersonal relations; social-cognitive and social-emotional functioning; and lifestyle and engagement. Each item starts with the stem: *With my increasing age, I realize that…* An example item assessing perceived age-related gains is *With my increasing age, I realize that I have more experience and knowledge to evaluate things and people.* An example of item assessing perceived age-related losses is *With my increasing age, I realize that I have less energy.* Respondents rate how much each item applies to them on a five-point Likert scale (1 = not at all; 2 = a little bit; 3 = moderately; 4 = quite a bit; 5 = very much). Higher scores indicate higher perceived age-related gains and losses, respectively (range: 5—25). Both subscales were found to have good internal reliability in several countries with Cronbach’s alphas consistently above 0.70 for perceived age-related gains and above 0.79 for perceived age-related losses (e.g., [16, 102]).

*Felt age* (FA) will be assessed with a single-item question adapted from the National Survey of Midlife development in the United States (MIDUS) [15] asking participants to write the age (in years) that they feel most of the time. A proportional discrepancy score will be calculated by subtracting the participants’ chronological age from their felt age, and by dividing this difference score by participants’ chronological age. A positive value indicates an older felt age, whereas a negative value indicates a younger felt age. Typically, in western countries the majority of older individuals feel between 13 and 18% younger than their age [103], and a younger felt age is used as indicator of more positive self-perceptions of aging [104].

*Subjective Nearness to Death* will be assessed with the one-item question: *I have a feeling that my life is approaching its end* [66]. Answer options range from 1 (not at all) to 5 (very much).

#### Self-reported physical activity

Self-reported physical activity will be assessed with the International Physical Activity Questionnaire short form and the Saltin-Grimby Physical Activity Level Scale. In the *International Physical Activity Questionnaire* (IPAQ) [67] short form participants are asked to refer to the past seven days and to report how much time they spent sitting during a day; how many days they walked for at least ten minutes at a time; how many days they did moderate physical activities like gardening, cleaning, and bicycling; and how many days they did vigorous physical activities like heavy lifting, heavier garden or construction work, and aerobics. Separate scores can be obtained for walking, moderate intensity physical activities, and vigorous physical activities as follows: (1) Walking metabolic equivalent of task (MET)-minutes/week = 3.3 * walking minutes * walking days. (2) Moderate MET-minutes/week = 4.0 * moderate-intensity activity minutes * moderate days. (3) Vigorous MET-minutes/week = 8.0 * vigorous-intensity activity minutes * vigorous-intensity days. The International Physical Activity Questionnaire total score derives from the sum of the scores for walking, moderate-intensity, and vigorous-intensity activities.

The *Saltin-Grimby Physical Activity Level Scale* [68, 69] consists in a single-item question asking participants to report on average how much they moved during their leisure time. At baseline participants will be asked to answer thinking about the past 12 months whereas at follow-ups they will be asked to think about the past three months. To answer, participants can choose from four categories: 1 = physically inactive, 2 = some light physical activity, 3 = regular physical activity and training, and 4 = regular hard physical training for competitive sports. Participants are instructed to choose the option physically inactive when they have been almost completely inactive, reading, watching television, watching movies, using computers or doing other sedentary activities, and during leisure time. Participants are instructed to choose the option some light physical activity when they have been physically active for at least four hours/week, such as riding a bicycle or walking to work, walking with the family, gardening, fishing, table tennis, and bowling. Participants are instructed to choose the option regular physical activity and training when they have spent time doing heavy gardening, running, swimming, playing tennis, badminton, calisthenics and similar activities, for at least two–three hours/week. Participants are instructed to choose having done regular hard physical training for competitive sports when they have spent time running, orienteering, skiing, swimming, playing football, handball etc. several times per week.

### Secondary intervention outcomes

#### Ageism

The *World Health Organization-Ageism Scale* will be used to measure ageism in this study, including both experienced ageism and other-directed ageism [70]. *Experienced ageism* will be assessed with 15-items covering self-directed ageism: self-stereotypes (two items), self-prejudice (one item), self-discrimination (two items; interpersonal ageism: interpersonal stereotypes (two items), interpersonal prejudices (two items), interpersonal discrimination (three items) and institutional ageism: institutional discrimination (three items). A sample item is *Due to my age, I limit my participation in discussions even when they are about things that affect me.* Answer options for all items are 1 = strongly disagree; 2 = disagree; 3 = neither agree nor disagree; 4 = agree; 5 = strongly agree. The total score is obtained from the sum of items scores. Higher scores indicate greater ageism. Importantly, as mentioned in the introduction, self-directed ageism and self-perceptions of aging are conceptually different and hence they will be assessed with different measures in this study.

*Other directed-ageism* will be assessed with 23 additional questions developed by the Word Health Organization [70]. A sample item is *Older people lack purpose in life*. Answer options for all items are 1 = totally agree; 2 = agree; 3 = neither agree nor disagree; 4 = disagree; 5 = strongly disagree. The total score is obtained from the sum of items scores. Higher scores indicate less other-directed ageism. For both self-directed and other-directed ageism participants will be instructed to answer questions thinking about the last 12 months at baseline and thinking about the last three months at post-intervention and follow-up assessments.

#### Subjective views of couple's joint aging

The 14-item *Subjective Views of Couple's Joint Aging* (SVoCJA) scale [71] will be used to assess positive and negative subjective views of couple’s joint aging. Seven items capture positive subjective views of couple’s joint aging and a sample item is *the thought that our time together is limited will make us want to love each other more.* The remaining seven items capture negative subjective views of couple’s joint aging and a sample item is *Dealing with health issues will take a heavy toll on our relationship*. Each item is answered on a five-point Likert scale: 1 = strongly disagree; 2 = slightly disagree; 3 = neither agree nor disagree; 4 = slightly agree; 5 = very much agree**.** Subscales scores are obtained by summing up the scores of their respective items. In the validation sample, Cronbach’s alpha coefficients were 0.77 for positive and 0.78 for negative subjective views of couples’ joint aging.

#### Anxiety and depressive symptoms

Anxiety and depressive symptoms will be assessed with the four-item version of the *Patient Health Questionnaire* (PHQ-4) [72], which assesses core symptoms of depression and anxiety over the past two weeks. A sample item is *Over the last two weeks, how often have you felt little interest or pleasure in doing things?* Answer options are on a four-point Likert scale: 1 = not at all; 2 = several days; 3 = more than half the days; 4 = nearly every day. The total score is obtained from the sum of items scores. Higher total scores (range: 4—16) indicate more non-specific anxiety and depressive symptoms. Cronbach’s alpha was above 0.80 for both depression and anxiety subscales in the validation sample, indicating good internal reliability [72].

#### Physical fitness

Objective indicators of physical fitness will be body mass index, grip strength, waist circumference, and the Senior Fitness Test. *Body Mass Index* will be calculated from self-reported weight and height (weight in kg/height in m^2^) [105]. Waist circumference will be assessed by the researcher during the baseline, post-intervention, and six-month in person follow-up assessments using a flexible measuring tape [106]. The measure will be taken at midpoint between the lower margin of the last palpable rib and the top of the hip bone.

*Grip strength* [74] will be measured with a dynamometer and coded as a continuous variable*.* The *Senior Fitness Test* [73] comprises six tests. First, the Chair Stand Test requires participants to repeatedly stand up from and sit down on a chair for 30 s. The number of stands is recorded and reflects lower body strength. Second, the Biceps Curl Test requires participants to repeatedly lift a five lb (2.27 kg) weight (for women) or an eight lb (3.63 kg) weight (for men) for 30 s. The number of lifts is recorded and reflects upper body strength. Third, the Six-Minute Walk Test asks participants to walk for six minutes on a straight line. The distance (m) walked is recorded and reflects aerobic endurance. Fourth, the Chair Sit and Reach Test asks participants to reach forward toward the toes by bending at the hip while being seated. This task is measured in distance (cm) and reflects lower body flexibility. Fifth, the Back Scratch Test asks participants to start from standing position, place one hand behind the head and back over the shoulder and reach as far as possible down the middle of the back and place the other arm behind their back and reach up as far as possible attempting to touch or overlap the middle fingers of both hands. This test is measured in distance (cm) and reflects upper body flexibility. Finally, the 2.45—m Up-and-Go Test asks participants to stand up from a chair and walk as quickly as possible around a cone, and to return to the chair to sit down. This test is measured in time (seconds) and reflects agility and dynamic balance.

#### Cognitive functioning

This study will assess aspects of executive function. cognitive flexibility; attention; working memory; and inhibition.

Cognitive flexibility and visual attention will be assessed with the paper and pencil version of the *Trail Making Test* [75], which comprises two parts. Trail Making Test-A requires participants to draw lines sequentially connecting 25 encircled numbers distributed on a sheet of paper. Task requirements are similar for Trail Making Test-B except the participant must alternate between numbers and letters (e.g., 1, A, 2, B, 3, C, etc.). For both Trail Making Test A and B the total score is the amount of time required to complete the Trail Making Test A subtracted from the amount of time required to complete the Trail Making Test B. Working memory will be assessed with the paper and pencil version of the Digit Span Forwards and Backward tasks taken from the Wechsler Adult Intelligence Scale [76], the PMR Phonological Verbal Fluency test, and the Animal Naming Fluency test [77]. In the *Digit Span Forward* [76], participants are asked to repeat digits forward (14 number sequences ranging from three to nine digits). Each correctly recalled sequence is scored as one point (possible total score range: 0—14). Higher scores indicate greater working memory. In the *Digit Span Backward* participants are asked to repeat digits backward (14 number sequences that range from five to eight digits in length). Each correctly recalled sequence is scored as one point (possible total score range: 0—14). Higher scores indicate greater working memory. In the *PMR Phonological Verbal Fluency Test* participants are asked to generate within 60 s as many words as possible that start with the letters P, M, and R. For each letter the number of correct words is registered, excluding intrusions and perseverations. This task also assesses executive functions in addition to working memory. In the *Animal Naming Fluency Test* participants are asked to generate as many animal names as possible within 60 s. The number of correct words is registered, excluding intrusions and perseverations. Inhibition will be assessed with the *Stroop Color-Word Test* [78]. In this test, participants are shown a list of color words, such as “red,” “blue,” “green”, printed in different-colored inks. The ink color may match the word (i.e., congruent condition) or differ from the word (i.e., incongruent condition). Participants are instructed to name the ink color of each word as quickly and accurately as possible, ignoring the written word itself. To assess attention, the D2 test will be used [79]. The D2 test is a paper-and-pencil cancellation task consisting of 14 rows of visually similar stimuli containing the letters *d* and *p*, each marked with one to four dashes positioned above and/or below the letter. Participants are instructed to scan each row from left to right and cross out only target stimuli, defined as the letter *d* marked with exactly two dashes, while ignoring all distractors. Each row has to be completed within a time limit of 20 s. The total score is obtained by calculating the total number of items processed within timed rows and subtracting errors (i.e., missed targets or incorrectly marked non-targets). A composite score for executive function will be created by summing up standardized scores on the Trail Making Test [75], the Digit Span Backward task [76], the PMR Phonological Verbal Fluency test, the Animal Naming Fluency test [77], the Stroop Color-Word Test [78]; and the D2 test [79].

### Other variables

#### Sociodemographic questions

Sociodemographic questions will comprise age in years; sex; gender; sexual orientation; ethnic origin; highest level of education achieved and years of education; marital status; employment status; and total income (this last question will be optional). Participants will also fill the *MacArthur’s Scale of Subjective Social Status* [93]. More specifically, participants will be presented with a graphical ten-rung ladder representing “where people stand in Spain”, with the top rung representing the best off, and the bottom rung representing the worst off, in terms of education, money, and jobs in Spain. Participants will indicate where they think they stand at that time in their lives, relative to other people in Spain. Higher scores indicate higher subjective social status.

#### Physical health

Participants will be presented with a list of 21 *chronic physical health conditions* such as stroke and diabetes and asked to self-report which conditions they have. A count score will be created for the total number of chronic physical health conditions participants have.

#### Mental health

Participants will be presented with a list of 15 *mental health conditions* such as depressive disorders and anxiety disorders and asked to self-report which conditions they have. A count score will be created for the total number of mental health conditions participants have.

#### Personality

The *Ten-Item-Personality Inventory* [94] will be used to assess five personality traits: extraversion, agreeableness, openness, conscientiousness, and neuroticism. Each personality trait is assessed with two items. A sample item assessing extraversion is *I see myself as extraverted, enthusiastic.* A sample item assessing agreeableness is *I see myself as critical, quarrelsome.* A sample item assessing conscientiousness is *I see myself as dependable, self-disciplined.* A sample item assessing neuroticism is *I see myself as anxious, easily upset.* A sample item assessing openness is *I see myself as open to new experiences, complex.* For each item, participants select one of the possible seven answer options: 1 = disagree strongly; 2 = disagree moderately; 3 = disagree a little; 4 = neither agree not disagree; 5 = agree a little; 6 = agree moderately; and 7 = agree strongly. Scores for each personality trait are obtained by summing up their respective items (possible range: 2—14). Higher scores indicate greater presence of a given personality trait.

#### Couple dynamics

Quality of the relationship with one’s partner will be assessed with the six-item *Quality of Marriage Index* [91]. This questionnaire asks participants to report the extent to which they agree or disagree with global statements regarding the quality of their relationship. A sample item is *We have a good marriage*. Items from one to five are answered on a seven-point Likert scale (1 = strongly disagree to 7 = strongly agree) whereas item six is answered on a ten-point Liker scale (1 = extremely low to 10 = extremely high). The total score is obtained from the sum of items scores. Higher scores reflect better marital quality (possible range: 6—45).

*Dominance* will be assessed using a single-item question: *In your current romantic relationship, which one of you is more dominant/powerful?* [92].

Participants will also be asked how many years they have been with their partner, whether they have children, and whether they have grandchildren.

#### Self-perceptions of aging

The Subjective Aging Perception Scale (SAPS) [88, 89] will be used at baseline assessment only to assess convergent validity of the Awareness of Age-Related Change scale. The scale assesses self-perceptions regarding four domains: physical self-concept, cognitive self-concept, subjective perception of time, and subjective perception of social relations, and each concept/subscale is composed of three items. A sample item for the physical self-concept is *I think I am pretty fit for my age.* A sample item for the cognitive self-concept is *I think I have the same mental agility as before.* A sample item for the subjective perception of time subscale is *I am so busy that there just aren’t enough hours in the day.* A sample item for the subjective perception of social relations subscale is *I often get bored.* Participants respond to each item on a seven-point Likert type scale (from 1 = totally disagree to 7 = totally agree). Subscales scores are obtained by the addition of each of the items included in the given subscale. The total score is obtained by adding up the results for each subscale (possible range: 12—84). Higher scores indicate more positive self-perceptions of aging. In the Spanish validation of the Subjective Aging Perception Scale, Cronbach’s alphas for all scales were above 0.91, indicating excellent internal reliability.

#### Ageism

The *Expectations Regarding Aging* (ERA) questionnaire [90] will be used at baseline assessment only to test convergent validity of the *World Health Organization-Ageism Scale.* The Expectations Regarding Aging scale is the only ageism measure that was found to meet minimum requirements for psychometric validation [107] and that is therefore suitable for convergent validity purposes. The Expectations Regarding Aging questionnaire comprises three four-item subscales assessing different domains: physical health, mental health, and cognitive function. An example of item for the physical health scale is *When people get older, they need to lower their expectations of how healthy they can be*. An example item for the mental health scale is *I expect that as I get older, I will spend less time with friends and family*. An example item for the cognitive function scale is *I expect that as I get older, I will become more forgetful.* For each item respondents choose an answer option from 1 (*definitely true*) to 4 (*definitely false*). Higher subscale scores indicate more positive expectations regarding aging (possible range: 4—16). A total score can also be calculated by summing subscales scores. In the validation sample of the Expectations Regarding Aging questionnaire, Cronbach’s alpha exceeded 0.75 for each subscale and equated 0.89 for the expectations regarding aging total score, indicating excellent internal reliability [90].

### Daily physical activity

During the 12-week intervention only those participants who undertake the intervention will be instructed to record in *ad-hoc paper and pencil daily diaries* whether they walked and how many kilometers they walked that day. In the same daily diaries participants will also be instructed to rate the difficulty of the physical activity they did using the *Borg Rating of Perceived Exertion Scale* [86]. For this scale scores can range between six and 20. Lower scores indicate lighter physical exercise whereas higher scores indicate harder physical exercise. Daily diaries will be used to cross-check participants answers to the physical activity questionnaires and the results of data analyses. However, this variable will not be used as intervention outcome as only those who undertake the intervention will complete the daily diaries whereas the control group and one partner from the single-partner intervention group will not be instructed to complete the daily diaries.

### End-of-intervention questionnaire

At the end of the last intervention session all participants will complete, using pen and paper, a questionnaire comprising 18 multiple answer and open-ended questions. These will assess perceived efficacy of the intervention, enjoyability of the intervention, and couple dynamics. The full list of questions can be found in Open Science Framework [101].

### Interviews

In the two weeks following the end of the in-person psychoeducational intervention, ten couples from the dual-partner intervention group and ten couples from the single-partner intervention group will be randomly selected for online interviews via Phone/Teams/Zoom. Partners will be interviewed separately. Those participants who will have undertaken the intervention will be asked to further elaborate on their answers on the end-of-interview questionnaire. Those who will not have undertaken the intervention will be asked whether and how changes in their partner affected them. The full list of questions can be found in Open Science Framework [101].

### Intervention

#### Psychoeducational component targeting self-perceptions of aging

The psychoeducational component of the intervention was developed by Wolff, Warner [50] in 2013—2014 and further refined by Beyer, Wolff [53] in 2017—2018 and proved effective at trial level in German older adults aged 66—88 years. In this project the psychoeducational component will be delivered through three in-person one-hour sessions during weeks one, three, and five of the 12-week intervention. The psychoeducational sessions will be delivered by two psychologists that will be different from the researchers/psychologists who will assess participants at baseline and follow-ups. The psychoeducational component will include homework between sessions two and three. To ensure comparability of content and procedure for all intervention groups, a modified version of Beyer and colleagues’ 2019 intervention manual adapted for the Spanish population will be used. The first psychoeducational session will consist of a quiz on aging with questions about healthy aging and aging in general to detect own negative self-perceptions of aging as well as misconceptions regarding age and aging (see Table 2 for description of intervention content). Possible false beliefs will be corrected by providing information on empirical findings during the discussion of the answers. The second psychoeducational session will occur three weeks later, and it will provide information on benefits of more positive self-perceptions of aging regarding health, functional and cognitive abilities, as well as longevity. It will also include a group discussion on these benefits. Additionally, participants are given homework for the next intervention session. As part of the homework participants will be asked to observe themselves or others to detect negative age stereotypes or self-perceptions of aging in everyday life and to write down the situations and the negative self-perceptions of aging on a worksheet. During the third psychoeducational session, the homework will be presented and discussed in the group. Subsequently, a technique to change negative automatic thoughts about aging [97] will be taught using a hypothetical situation: *Imagine you are crossing the road at the traffic lights. Before you get to the other side, the traffic lights turn red again. A group of young people is waiting on the other side of the road and starts laughing. You might automatically think “The young people are laughing at me because I’m old and slow”*. The technique consists of three steps: (1) sensitizing and increasing awareness of one’s automatic and negative thoughts about aging, (2) questioning these thoughts and, (3) in the case of too negative thoughts, replacing them with more realistic, neutral, and/or more positive ones. To achieve steps two and three, participants will be asked to find some alternative, positive or neutral, and conceivable interpretations of the situation described. An example of alternative thought for the above-reported example could be: *The young people were talking about another topic and didn’t laugh at me,* or *the time was too short before the traffic lights turn red.* In class, participants will then be asked to apply this technique to their own situations recorded in their homework. Finally, the last session will end with a group discussion and by asking participants to write down some positive views on aging which are subsequently discussed in the group.

#### Behavioral component promoting physical activity

The behavioral component of the intervention aims to promote walking three to five times per week and is based on the Spanish *Projecte Moviment* [80, 108]. It will last 12 weeks as this should be sufficient enough to lead to a significant increase in participants’ physical activity and cardiorespiratory fitness [80], and it should also be long enough for physical activity to become an habit [109]. The walking program is based on international guidelines of physical exercise [110]. Participants will be instructed to walk briskly in one continuous bout (45 min for five days, 225 min per week). Intensity, duration, and times per week will be initiated in a stepwise manner to reduce the possibility of injury. During the first week participants will be asked to walk 30 min at 9–10 on the Borg Rating of Perceived Exertion Scale (i.e., light intensity) [86]. During the second week, participants will be asked to walk 45 min with the same intensity. During the remaining weeks, participants will be asked to maintain the duration of 45 min and increase the intensity of the activity to a moderate-high effort that corresponds with 12–14 in the Borg Rating of Perceived Exertion Scale. The first day of the psychoeducational intervention participants will be trained to use the Borg Rating of Perceived Exertion Scale [86] and to record this intensity and frequency of activity in a paper daily diary. To ensure adherence to the protocol, every two weeks the project team will contact participants via text message or email to remind them about their involvement in the study and to record their walking sessions in the daily diary.

#### Safety considerations

To anticipate, prevent, and answer medical or personal issues raised by participants, the project team will take into account several considerations. First, during an initial screening done via phone, only those participants who are physically fit to start a walking intervention will be allowed to take part in the study (i.e., participants that do not self-report mobility issues and that during the baseline physical fitness assessment do not show any physical concerns/limitations). Second, all the baseline and follow-up assessments will be reviewed by the research team before allocating participants to intervention group to ensure safety during the intervention. Eventual abnormalities identified will be reported and these participants will be directed to corresponding healthcare service. Instructions for the behavioral component of the intervention include health advice to prevent injuries. Third, participants can contact via email or phone the research team for any problems or pain that they may experience. Again, participants will be redirected to corresponding healthcare services. Fourth, to ensure the health and wellbeing of participants throughout the study, every two weeks researchers will also contact participants via phone call, text message, or email to check whether they are experiencing any physical issues, pain, or if any adverse events occurred while exercising. In case of adverse events or in case of experiencing pain or other physical issues, participants will be contacted via phone by a member of the research team and encouraged to see a doctor to seek advice on whether they can continue with the behavioral intervention. In parallel, a member of the team, who is a physician, will be consulted. Adverse events and any related abnormality or interruption of data collection will be recorded in the dataset.

### Data quality

A computerized database will be used to collect and organize all data. Data will be collected without personal identifying information using a code assigned by the researchers. Only the core research team will have access to this information in case of an incident. Data from all participants will be collected/used regardless of whether the participant withdraws from the intervention or not (unless the participant retrospectively asks the study team to destroy their data). All assessments and databases will be double-checked. The study team will follow Data Quality Assessment Checklist and Recommended Procedures that assess a variety of dimensions as validity, reliability, timeliness, precision, and integrity. The study team will analyze whether participants’ data is related to expected physical changes. If the study team identifies any issues in the data, they will inform and apply any required statistical procedures to control them. No formal data monitoring committee nor external auditing is planned due to the small scale and non-commercial nature of this study. However, the study team will implement quality checks to ensure compliance with the study protocol. More specifically, the study team will periodically review consent forms, completeness of online questionnaires, randomization, and participants’ safety. These internal checks will be documented.

### Statistical analyses

This intervention is part of a broader study/project investigating “Self-perceptions of aging in older couples”. Description of this broader study, including details on data management and data storage are reported in Open Science Framework [101].

To address the first and second study hypotheses, pre-post intervention and follow-up changes in intervention outcomes in the dual-partner intervention group, single-partner intervention group, and control group will be investigated using mixed effects models using random slopes to allow for individualized change over time. More specifically, to address the first research question we will compare participants from the dual-partner intervention group and single-partner intervention group versus those from the control group. To address the second research question, we will compare participants from the dual-partner intervention group with those of the single-partner intervention group. To address the third study hypothesis, we plan to fit latent change score models for the entire sample. That is, we will take all the 180 study couples and estimate their individual changes (in a specific variable) as well as the covariance/correlation of these individual changes. This will make it possible to get an initial idea of whether change in primary and second intervention outcomes is interrelated (independently of whether both partners received the intervention, one partner received the intervention, none of the partners received the intervention). In a subsequent step, we will then add to the model a grouping variable (comprising the following categories: single-partner intervention group, dual-partner intervention group, or control group) and investigate whether this grouping variable predicts the covariance/correlation in individual change. In line with the study hypothesis that the intervention is more effective when delivered to both partners compared to one partner alone, we expected that the dual-partner intervention group will show a significant covariance and that this covariance is higher than the one in the control group and in the single-partner intervention group.

In all the models, age, sex, education, month when the intervention will be undertaken, delivery batch, will be included as covariates. In the models with physical activity as outcome, baseline physical activity will also be included as covariate. Length and quality of the relationship and cohabitation status will be explored as potential covariates for the first and second study hypotheses. To address the third hypothesis, length and quality of the relationship, cohabitation status, dominance of the partner, and personality will be considered as additional covariates in the models. In the analyses, secondary outcomes will be treated as exploratory. For the six primary outcomes (see Table 1) it is instead planned to use False Discovery Rate correction [111] to adjust for multiple comparisons. Intention-to-treat analysis will be performed, which means that data of all trial participants in the groups to which they were randomized (i.e., after the collection of baseline data) will be processed, regardless of whether they received or adhered to the allocated intervention. It is assumed that most participants in the first and second groups/arms will receive all intervention sessions.

For study analyses, assuming that data is missing at random, missing values will be imputed using multiple imputation by chained equations; 25 imputed datasets will be generated. We will check for assumptions related to missing data. First, we will describe patterns of missingness across study variables, intervention groups, and timepoints (pre-intervention, post-intervention, three-month follow-up and six-month follow-up assessments). Second, we will model observed covariates of missingness. Identification of significant covariates will imply that data are not missing completely at random and that missing at random is therefore plausible. Third, to inform about attrition, we will compare the descriptive statistics of participants who have completed all the follow-ups with those who did not.

Additionally, a per-protocol analysis of the participants who completed the study/intervention without major protocol violation (e.g., who attended at least two of the three psychoeducational sessions and who reported having engaged in physical activity at least 80% of the expected times) will be performed. The per-protocol analysis will be performed as secondary analysis, if there are enough participants in the three intervention groups/arms, who are not lost to follow-up assessments and if there are enough participants in groups one and two who undertook the entirety of the intervention. Quantitative analysis will be conducted using STATA version 19, Mplus, and R. Analyses for the first two research questions will be conducted in STATA and Mplus whereas analyses for the third research question will be conducted in R and Mplus. Content analysis [112] will be used to analyze data from the open-ended questions and from the interviews conducted at the end of the intervention. These analyses will be conducted using Atlas.ti by two independent researchers.

### Dissemination policy

The dissemination plan for this study is reported in Open Science Framework [101]. For any manuscript/publication resulting from this study authorship will be decided based on the International Committee of Medical Journal Editors (ICMJE) [113]. That is, individuals, will be added as authors when they (1) provide a substantial contribution to conception or design of the work, or acquisition, analysis, or interpretation of data; (2) draft or critically revise the work for important intellectual content; (3) provide approval of the final version of the manuscript to be published; (4) are accountable for all aspects of the work. Those who will not meet all these criteria will be listed in the acknowledgments of publications resulting from this project.

## Discussion

Given the growing proportion of older people worldwide [1], the promotion of active and healthy aging are key priorities. Fostering positive self-perceptions of aging may help in this regard. Indeed, positive self-perceptions of aging are associated with engagement with health-enhancing behaviors and of better scores on a wide range of indicators of mental, physical, and cognitive health in older age [13, 23, 30, 114]. Despite the wide amount of evidence supporting the longitudinal associations between self-perceptions of aging and health outcomes, intervention programs promoting positive self-perceptions of aging and proving effective at trial level are still relatively few [45, 46]. In addition, all existing interventions promoting positive self-perceptions of aging have so far been delivered at the individual level [46]. However, individuals’ self-perceptions of aging may be influenced by the beliefs that people close to them have about aging [54–56] and by how they are treated by others during daily interactions and conversations [28, 51, 57, 58]. Indeed, research studies have shown that spouses/partners tend to have similar self-perceptions of aging and to influence each other’s self-perceptions of aging over time [54, 56, 60–62]. So, if the aim is to change how older people think about themselves, it may also be necessary to modify how they are perceived and treated by relevant others.

This pre- post-intervention study will investigate for the first time whether an intervention aiming to promote positive self-perceptions of aging and physical activity in older people is more effective when both partners undertake it (dual-partner intervention group) compared to when only one partner undertakes it (single-partner intervention group). Using data from the single-partner intervention group, the study will also investigate for the first time whether, when only one partner undertakes the intervention, changes in primary and secondary outcomes occur to some extent also in the other partner who does not undertake the intervention. The multicomponent intervention that will be used in this project is based on a psychoeducational intervention previously validated in Germany [50, 53] and on a physical activity/walking intervention previously validated in Spain [80, 108]. This project will therefore test for the first time the efficacy of these two components joint together. This project will also observe what type of changes the multicomponent intervention may produce in couples looking at self-perceptions of aging and physical activity as primary outcomes, and at ageism, subjective views of couple’s joint aging, perceived age-related changes in one’s partner, anxiety and depressive symptoms, physical fitness, and executive function, and attention as secondary outcomes. To our knowledge, this is the first intervention targeting positive self-perceptions of aging that integrates a dyadic design. This approach acknowledges the interpersonal nature of belief formation and maintenance in later life, and may represent a paradigm shift in behavioral aging interventions.

It is expected that results will support the additional benefit of targeting couples rather than single individuals. This hypothesis is based on previous evidence on physical activity interventions showing that participating to the intervention with a partner increases intervention outcomes [82, 114]. It is also likely that this study will initiate a novel thread of research testing the efficacy of interventions promoting self-perceptions of aging, but also physical activity, in other types of dyads and small groups. Indeed, while this study will specifically target couples/partners, future research studies could focus on friends or intergenerational groups such as families comprising children, parents, and grandparents. It is also expected that as research in this emerging field will grow, models of self-perceptions of aging will be revisited to consider the interpersonal realm. Finally, it is expected that this study will lead to clinical implications for other types of dyads such as caregiver care-receiver dyads. Indeed, it has previously been found that caregivers and care-receivers (e.g., people with dementia) have similar self-perceptions of aging, and that more positive self-perceptions of aging in the caregiver are related to greater well-being and life satisfaction in the care-receiver [115].

Nevertheless, the proposed study has limitations. First, participants will not be blinded as they will know in which intervention group they will be assigned. However, the researchers who will assess participants will be blinded. Second, study participation is demanding for participants as, in addition to completing baseline assessment and three follow-up assessments (including two undertaken in-person), they will be asked to adhere to a 12-week intervention comprising both a psychoeducational component delivered as three in-person sessions and a behavioral component implying independent physical activity/walking up to five times per week. This may result in low adherence. Intention to treat analyses and per protocol analyses will help to describe discrepancies and control attendance. Moreover, several strategies will be adopted to increase adherence including reminding participants via calls or text messages of intervention sessions and assessments, recognizing reached milestones (e.g., first week of walking completed, one month of walking completed, etc.) via text messages, giving participants the opportunity to choose between multiple timeslots for in-person assessments, encouraging peer interaction during the psychoeducational sessions, giving participants a certificate of completion of the intervention at the end of it, and payment at the end of the study. Third, although the planned sample size is adequate to address the main study questions, it is unlikely that it will make it possible to explore additional questions such as investigating the moderating roles of length of relationship, quality of the relationship, age-difference between partners, dominance, gender, and personality when looking at the associations between the changes in the study outcomes of one partner with the changes in the study outcomes of the other partner (single-partner intervention groups). Fourth, study participants will have to complete some questionnaires online using a smartphone, tablet, laptop, or computer. Although technology illiteracy is not an exclusion criterion to this study as the researchers will be available to teach participants how to use technologies and fill questionnaires online, older adults without technology literacy may be reluctant from taking part in the study, and hence they may not be well represented in the study sample. Fifth, internet access is an inclusion criterion to this study. Older individuals who do not have internet at home or in a place nearby their house may not be represented in the study. Sixth, as data collection will start each time a group of about 42 participants will be recruited, the intervention will be delivered at different times for different participants. This is a limitation as different periods of the year may influence participants’ motivation to undertake physical activity. Moreover, it is possible that the researcher who will deliver the intervention improves their ability to deliver the intervention, and this may impact on the efficacy of the intervention. However, in the analyses we will control for this variable. Seventh, assessment of physical activity will be self-reported. This is a limitation as this type of assessment can be subject to both recall bias and social desirability [116]. However, tools for objective assessment of physical activity, such as the use of wearables, can be subject to low maintenance/adherence [117, 118]. Eight, as the psychoeducational intervention will be delivered in a group setting, we cannot exclude that eventual changes detected in intervention outcomes are not due to social and engagement factors, rather than to the psychoeducational content itself. To address this issue, future research could therefore employ an active control condition to better isolate the specific contributors to changes in intervention outcomes.

## Supplementary Information

Supplementary Material 1.

Supplementary Material 2.

## Data Availability

This project will be based on newly collected data which will be made publicly available with no limitations, together with metadata, via Open Science Framework once all study analyses will be concluded and published. Inquiries regarding data can be directed to the corresponding author. For information on the material of the psychoeducational intervention please contact Dr Serena Sabatini (serenasabatini@ub.edu) and Prof Susanne Wurm (Susanne.Wurm@med.uni-greifswald.de.) For information on the material of the behavioral component please contact Prof Maria Mataro Serrat (mmataro@ub.edu).
