# Supplementary material for: Reframing Expectations about aging – Physical Activity and Inclusive Reappraisal (RE-PAIR): Protocol of a randomized intervention promoting positive self-perceptions of aging and physical activity in older couples
Source: BMC Geriatr. 2026 May 14;26:931. doi: 10.1186/s12877-026-07603-7 (PMC13348951; doi:10.1186/s12877-026-07603-7)
Supplement: Supplementary file 1 — Supplementary Material 1. [file 12877_2026_7603_MOESM1_ESM.docx]

**Supplementary Text**

**Phone-based screening questions**

Good morning/afternoon. I am [researcher’s name]. Thank you for your interest to take part in the study “Views on Aging and Physical Activity in Couples Aged 65 and Over”. The purpose of this quick call is to check whether you are eligible to take part in the study, that is to make sure you match the characteristics of the people we would like to take part in the study. I will ask you a few simple questions. It should take around 10-15 minutes.

1. First of all, may I ask your name and surname? [keep note]
2. Do you speak Catalan or Castilian Spanish? [candidates who speak Castilian Spanish or Catalan are eligible to take part in the study]
3. Are you married or do you have a partner with whom you co-habit or with whom you have been together for at least one year and with whom you meet at least twice a week? [if not, thank the candidate but tell them they are not eligible to take part in the study]
4. Does your partner agree to take part in the study? [if not, thank the candidate but tell them they are not eligible to take part in the study. If they do not know because they have not yet consulted their partner, ask them to do so and let us know via email, phone call, or text message]
5. Where do you live? [candidates are eligible only if they live in Barcelona or in towns nearby Barcelona]
6. How old are you and how old is your partner? [candidates are eligible to take part in the study if they and their partner are aged 65 years of over]
7. Have you ever received a diagnosis of neurodegenerative disorders such as dementia? [if they have dementia or mild cognitive impairment, candidates are not eligible to take part in the study]
8. Do you currently have a diagnosis of mental illness that is significantly impacting your daily functioning, that is your ability to undertake daily chores, work, or social interactions? [if yes, candidates are not eligible to take part in the study]. However, individuals with a current diagnosis of depression or anxiety that is managed can take part in the study.
9. Are you currently abusing of alcohol or drugs or have you abused of alcohol or drugs in the past five years? [if yes, candidates are not eligible to take part in the study]
10. Do you have any physical limitations that impair you from starting a physical activity intervention consisting in walking between three to five times per week and to do some physical tasks such as stand up from and sit down on a chair, lift a weight of about 2.27 kg, walk for 6 minutes. If you are unsure whether you are fit enough to start exercising, we advise asking advice to your general practitioner / family doctor. [if candidates are not able to give an answer, tell them they can contact their doctor and let the research know by emailing at sabatiniserena@ub.edu]
11. Do you have access to a smartphone/tablet/laptop/computer with internet access? This could even be the device of a child, grandchild, friend or neighbour that allows you to use it to complete some questionnaires. If you are not a good tech user, do not worry. We will show you how to complete the questionnaires online. [if they do not have access to a device with internet, they are not eligible to take part in the study].
12. 22-point telephone Adult Lifestyles and Function Interview MMSE (ALFI-MMSE) At least score of 15 (or higher) to take part in the study].
